# Editorial Comments to Impact of Approach Routes in MRI‐Ultrasound Fusion Prostate Biopsy on Post‐Biopsy Complications: A Propensity Score‐Matched Analysis

**DOI:** 10.1111/iju.70321

**Published:** 2025-12-17

**Authors:** Sunao Shoji

**Affiliations:** ^1^ Department of Urology Tokai University School of Medicine Isehara Japan

**Keywords:** complications, prostate biopsy, transperial approach

With the recent introduction of magnetic resonance imaging–transrectal ultrasound (MRI–TRUS) fusion image‐guided prostate biopsy, the role of prostate biopsy has expanded beyond determining whether prostate cancer is present. Clinicians are now expected to more accurately evaluate each patient's cancer status, including the maximum Gleason score, which reflects tumor aggressiveness, the intraprostatic cancer location, and the extent of tumor involvement. MRI–TRUS fusion‐guided biopsy has demonstrated improved diagnostic accuracy for these parameters compared with conventional techniques [1, 2].

According to the European Association of Urology (EAU) Guidelines, a systematic review and meta‐analysis of MRI‐targeted biopsy reported that transperineal targeted biopsy shows higher sensitivity for detecting clinically significant cancer than the transrectal approach (86% vs. 73%), with an evidence level of 2 (EAU Guideline) [3]. Recently, techniques such as template‐free transperineal biopsy and freehand approaches—designed to obtain cores directly from the center of the target and avoid pelvic bone obstruction—have become increasingly common. Although the transrectal approach has long been favored for its accessibility, the adoption of freehand transperineal techniques has rendered the transperineal method comparable or superior to the transrectal route in targeting performance.

Acute prostatitis and rectal bleeding remain notable risks of prostate biopsy. These complications occur mainly after transrectal biopsy and may necessitate interventions such as sepsis management or endoscopic hemostasis. Although their incidence is low, prevention is essential. The authors compared complications following targeted biopsy combined with a 12‐core systematic biopsy and demonstrated both the safety and the cancer‐detection advantages of the transperineal approach [4]. Although this investigation is a single‐center retrospective analysis, similar outcomes are expected in future multicenter prospective studies.

In Western countries, office‐based transperineal biopsy performed under local anesthesia is becoming increasingly common. In Japan, however, prostate biopsy is still frequently conducted in hospitals under saddle block or spinal anesthesia. With continued education and broader dissemination of local anesthesia techniques, MRI–TRUS fusion‐guided prostate biopsy may become more widely implemented in outpatient settings. Wider adoption of MRI–TRUS fusion‐guided biopsy is likely to improve patient selection for active surveillance and focal therapy [5] and is expected to significantly influence future management trends in localized prostate cancer.

## Author Contributions

**Sunao Shoji:** contributed to the conceptualization of the study and the writing of the manuscript.

## Ethics Statement

The author has nothing to report.

## Consent

The author has nothing to report.

## Conflicts of Interest

Sunao Shoji is an Editorial Board member of the International Journal of Urology. To minimize bias, they were excluded from all editorial decision‐making related to the acceptance of this article for publication.

## Linked Articles

“Impact of Approach Routes in MRI‐Ultrasound Fusion Prostate Biopsy on Post‐Biopsy Complications: A Propensity Score‐Matched Analysis,” https://doi.org/10.1111/iju.70275.

## Data Availability

The author has nothing to report.
